# Cas9 deletion of lutein biosynthesis in the marine alga *Picochlorum celeri* reduces photosynthetic pigments while sustaining high biomass productivity

**DOI:** 10.3389/fbioe.2023.1332461

**Published:** 2024-01-11

**Authors:** Melissa Cano, Anagha Krishnan, Devin A. Karns, Maria A. Likhogrud, Joseph C. Weissman, Matthew C. Posewitz

**Affiliations:** ^1^ Department of Chemistry, Colorado School of Mines, Golden, CO, United States; ^2^ ExxonMobil Technology and Engineering Company, Annandale, NJ, United States

**Keywords:** pigment reduction, CRISPR/Cas9, lutein, *Picochlorum celeri*, biomass productivity

## Abstract

Domestication of algae for food and renewable biofuels remains limited by the low photosynthetic efficiencies of processes that have evolved to be competitive for optimal light capture, incentivizing the development of large antennas in light-limiting conditions, thus decreasing efficient light utilization in cultivated ponds or photobioreactors. Reducing the pigment content to improve biomass productivity has been a strategy discussed for several decades and the ability to reduce pigment significantly is now fully at hand thanks to the widespread use of genome editing tools. *Picochlorum celeri* is one of the fastest growing marine algae identified and holds particular promise for outdoor cultivation, especially in saline water and warm climates. We show that while chlorophyll *b* is essential to sustain high biomass productivities under dense cultivation, removing *Picochlorum celeri’s* main carotenoid, lutein, leads to a decreased total chlorophyll content, higher a/*b* ratio, reduced functional LHCII cross section and higher maximum quantum efficiencies at lower light intensities, resulting in an incremental increase in biomass productivity and increased PAR-to-biomass conversion efficiency. These findings further strengthen the existing strategies to improve photosynthetic efficiency and biomass production in algae.

## 1 Introduction

Marine microalgae convert carbon dioxide (CO_2_) into complex molecules and require mainly light, inorganic nutrients and seawater. They are promising candidates for various applications in the pharmaceutical and nutraceutical industries and represent an attractive renewable and sustainable alternative to fossil fuels (Dismukes et al., 2008; Hannon et al., 2010). *Picochlorum celeri* emerged as a promising candidate for outdoor cultivation, attaining the highest productivities among all marine strains tested in the U.S Department of Energy DISCOVR program to-date, with an average of ∼31 g m^−2^ day^−1^ over 4 months in 2020, including several peak days at > 40 g m^−2^ day^−1^ (Krishnan et al., 2021). This strain is halotolerant and was selected as the fastest growing alga from collections of natural waters under high irradiance/temperature pressure (Weissman et al., 2018). It is able to attain a ∼2 h doubling time when exposed to high light (≥1,000 µmol PAR m^-2^ s^−1^) (Cano et al., 2021). It has a diploid genome (Becker et al., 2020) that is relatively compact, as is observed in other *Picochlorum* species (Foflonker et al., 2015; Gonzalez-Esquer et al., 2018; Krasovec et al., 2018; Dahlin et al., 2019). These features make *Picochlorum celeri* a promising platform to push boundaries in strain engineering, as genome-editing tools such as CRISPR become mainstream for this alga (Krishnan et al., 2020). As we are just beginning to unveil the potential of this strain, much fundamental knowledge remains to be learned about its photosynthetic and metabolic features to further improve its productivities. Indeed, while high outdoor productivities have already been recorded for this alga (Krishnan et al., 2021), strain engineering will likely be necessary to approach the higher desirable threshold of 60 g m^−2^ day^−1^ to establish algal production as an economically competitive biomass resource.

Large-scale algae cultivation is often light-limited due to decreased light penetration in the depths of the water column, self-shading, and fluctuating light intensities throughout the day and year, all significantly impacting the productivity and economic viability of the process (Ooms et al., 2016; Khan, Shin, and Kim, 2018). Additionally, while estimates of the theoretical efficiency of conversion of absorbed light energy into biomass are around 10%–12% (Zhu, Long, and Ort, 2008; Blankenship et al., 2011), the actual photosynthetic efficiencies in natural environments are much lower (<3%) (Sheehan et al., 1998; Grobbelaar, 2000; Lee, 2001; Moheimani and Borowitzka, 2007; Long, Marshall-Colon, and Zhu, 2015; Ort et al., 2015; Weissman and Nielsen, 2016). This is mainly due to the light saturation of photosynthesis occurring at intensities >50% lower than the maximum solar irradiance (Burlew, 1953; Kok, 1953; Meyers, 1971; Radmer and Kok, 1977a; Melis, 2009). In other words, a primary inefficiency is due to the over absorption of photons which arrive faster than can be processed by the slower biochemical reactions. Consequently, photoprotective mechanisms occur to dissipate excess energy at higher light intensities. Often the onset and turning off of these are not in synchronization with light absorption, further decreasing photosynthetic efficiency. As it is necessary for mass algal cultures to be operated as dense cultures, large-scale cultivation of algae inevitably leads to a non-homogenous light distribution whether in photobioreactors or in open ponds. Cells exposed to the highest light intensities (at the surface of the pond or closer to the light) over absorb photons, saturating their photosynthetic electron transport (PET) chain and dissipating the excess energy as fluorescence and heat through photoprotective mechanisms (Müller et al., 2001) safeguarding the photosystems.

The pigment content of algae, and in particular of *Picochlorum celeri*, which can reach up to 13% of the total particulate organic carbon or 6% of the AFDW in dense cultures (Cano et al., 2021), represents a major limitation to achieve higher biomass yields. The total pigment content as well as their complex arrangement with protein arrays in large antenna collecting the light (or light harvesting complexes, LHC) while increasing the effectiveness of light capture, reduce the efficiency of its use by the algal culture. The large antenna size represents a selective advantage in a competitive natural environment when light is limited, but becomes a source of inefficiency in mass cultures. Thus, domestication of algal strains in large-scale cultivation systems for biotechnological applications will require strain engineering to bypass the limitations engendered in high-density cultures (Mussgnug et al., 2007; Ort and Melis, 2011).

Reducing pigments in the photosynthetic antenna and/or reducing reaction centers to increase the saturating irradiance is one possible strategy to improve photosynthetic efficiency in mass cultures (Weissman, 1978; Nakajima and Ueda, 1997; Neidhardt et al., 1998; Nakajima and Ueda, 2000; Melis, 2009; Ort and Melis, 2011; Weissman and Nielsen, 2016; Kirst et al., 2017; Negi et al., 2020; VidyaVani et al., 2023). The idea is not new (Kok, 1953; Radmer Richard and Kok Bessel 1977b) and has been used somewhat successfully to improve the solar-to-biomass conversion efficiency in mutants with truncated light-harvesting chlorophyll antenna (or *tla*) (Nakajima and Ueda, 1997; 2000; 1999; Polle et al., 2002; Polle, Kanakagiri, and Melis, 2003; Kirst et al., 2012; Jeong et al., 2017). In particular, mutants targeting genes of the chloroplast signal recognition particle (CpSRP) pathway affecting the assembly of LHC proteins display a smaller chlorophyll (Chl) antenna size, a higher Chl a/*b* ratio, as well as a higher light intensity for the saturation of photosynthesis (Kirst et al., 2012; Kirst and Melis, 2014; Jeong et al., 2017; Kirst et al., 2017; Krishnan et al., 2023). These concepts are actively being explored in microalgae and hold promising potential for verification in outdoor systems in the future.

Antenna complexes in most green algae are comprised of LHC proteins, Chl *a*, Chl *b,* xanthophylls (e.g., lutein, violaxanthin, and neoxanthin), and carotenes (principally β-carotene). They surround the photosystem (PS) cores, forming PS-LHC supercomplexes, to which they transfer the captured light energy to initiate photochemical reactions. The photon energy harvested by LHCs is directed towards a special pair of Chl *a* forming the reaction center where charge separation occurs. The photosynthetic reaction centers generally contain Chl *a* and β-carotene, while the adjacent core complex proteins contain β-carotene and lutein as their only carotenoids (Peter and Thornber, 1991; Bassi et al., 1993). As Chl *b* is only present in the peripheral antenna, its levels are usually correlated to the functional size of the antenna. The expression of chlorophyllide *a* oxygenase (CAO), which converts Chl *a* into Chl *b* thus regulating Chl *b* levels, has been linked to antenna size (Masuda, Tanaka, and Melis, 2003; Tanaka and Tanaka, 2011). It has also been targeted to manipulate LHCs to increase light capture (Friedland et al., 2019). In the process of reducing pigment absorption, it is however crucial that the two PSs remain balanced so that the trapping centers are maintained in the correct state and that both the electron transfer and the distribution of light energy are optimized. As a result, in natural environments, photosynthetic organisms adjust their pigment composition and antenna proteins to achieve balance in the turnover rate of the two PSs (Radmer and Kok 1977a).

Previously, we identified the main pigments in *Picochlorum celeri* to be Chl *a*, Chl *b*, lutein, β-carotene, canthaxanthin, violaxanthin, neoxanthin, zeaxanthin and antheraxanthin. Chl dominates the pigment composition accounting for ∼80–85% of the total pigment content while lutein is the major carotenoid, representing ∼55–65% of all carotenoids (Cano et al., 2021). Lutein is synthesized by the lycopene ε-cyclase (LCYe), which catalyzes the ε-cyclization of lycopene. It has been localized in the crystallized structure of the LHC in higher plants and is the only xanthophyll detected in the PSII core (Bassi et al., 1993; Kühlbrandt, 1994).

Most strategies used so far to improve the light-dependent reactions of photosynthesis have been targeting the main pigment, chlorophyll, and other elements of the PET (Walter and Kromdijk, 2022). However, the over absorption by carotenoids might also affect photosynthetic efficiency. Some carotenoids play a crucial role in photoprotective mechanisms, but their sustained effect beyond stress conditions (mainly fluctuating light and temperature) might reduce overall efficiencies, especially in controlled growth conditions where extreme environmental fluctuations do not happen. This approach has been exploited recently in *Nicotiana* by targeting NPQ mechanisms: the xanthophyll cycle was engineered to accelerate the interconversion of violaxanthin and zeaxanthin resulting in a 15% gain in biomass yields in fluctuating light (Kromdijk et al., 2016). In this study, we used genome-editing tools (CRISPR/Cas9) to eliminate Chl *b* and lutein in *Picochlorum celeri*. We generated a chlorophyll *b*-deficient strain (CAO), a lutein-deficient strain (LCYe) as well as a strain missing both pigments (CAO/LCYe) and evaluated their productivity in diel-dense culture conditions using an automated photobioreactor.

## 2 Methods

### 2.1 Replication and statistics

Data points represent the mean of replicates from culture vessels independently inoculated with error bars representing standard deviations. For the diel cycle-dense culture experiments, each data point represents the mean of replicates from daily sampling from a single culture vessel with error bars representing standard deviation. Two-tailed *t*-tests were calculated to determine statistical significance.

### 2.2 Strain generation: sgRNA generation, Cas9-RNP transformation and selection

Details about the sequences of sgRNAs and primers used in this study can be found in Supplementary Table S1. We designed and tested *in vitro* sgRNAs targeting the chlorophyllide *a* oxygenase gene (*cao*) and the lycopene epsilon-cyclase gene (*lcye*) of *Picochlorum celeri* as previously described (Krishnan et al., 2020). 100 pmol of sgRNA was combined with either 70 pmol of purified *Streptococcus pyogenes* Cas9-NLS protein from qb3 MacroLab (University of California, Berkeley) or 50 pmol of Cas9-NLS protein from MilliporeSigma (CAS9PROT, MilliporeSigma, United States) and incubated at RT for 20 min to form RNPs. RNPs were delivered into *P. celeri* by electroporation along with 4 μg of the linearized selectable-marker plasmid pGAPDHNAT (Supplementary Figure S1A) conferring resistance to clonNAT (nourseothricin) for single knockout mutants (Krishnan et al., 2020). For the double knockout mutants CAO/LCYe, the CAO strain was used as the background strain to perform a second gene-editing step of the *lcye* gene using the selectable-marker plasmid pGAPDHble conferring resistance to phleomycin (Supplementary Figure S1B). Single colonies appeared after 14 days on plates containing the appropriate antibiotic(s) (clonNAT 75 μg mL^−1^ or/and phleomycin 20 μg mL^−1^) and kept in a 33°C/1% CO_2_ chamber under ∼100 μmol m^−2^ s^−1^ photosynthetically active radiation (PAR) and were restreaked before PCR screening for gene insertions/deletions (Supplementary Figures S2–S4).

### 2.3 Growth conditions and sampling

Cultures were axenic in all experiments. Dense culture medium (Weissman et al., 2018) composed of 40 g L^−1^ Instant ocean salt, 15 mg L^−1^ FeSO4 • 7H_2_O (stock solution of 2 g L^−1^ made in 2.7 g L^−1^ EDTA), 313.8 μg L^−1^ MnSO_4_•H_2_O, 24.23 μg L^−1^ CoCl_2_•6H_2_O, 48.82 μg L^−1^ ZnSO_4_ • 7H_2_O, 2 μg L^−1^ CuSO_4_ • 5H_2_O, 6.81 μg L^−1^ Na_2_MoO_4_ • 2H_2_O, 89 mg L^−1^ KH_2_PO_4_, 436 mg L^−1^ urea, 4.4 μg L^-1^ cyanocobalamin (vitamin B12), 4.4 μg L^-1^ Biotin, 1.54 mg L^−1^ thiamine (vitamin B1) and 100 mg L^−1^ kanamycin was used for liquid culturing. For maintenance on solid media, the strains were cultured on QATM plates (Krishnan et al., 2021). Cultures were grown in a custom-built solar-simulating automated photobioreactor system (the ALGiSIM system, ALGi, Inc.) as described previously (Cano et al., 2021) at a constant temperature of 33°C in 500-mL glass bottles bubbled with 2% CO_2_/air balance at a flow rate of 400 mL min^−1^ helping maintain a constant pH at ∼7.2. The solar day used had a maximum irradiance of 2,400 µmoles PAR m^−2^ s^−1^ at mid-day and represents a simulated summer day in Mesa, Arizona, excluding cloud cover, fog, rain, or other dynamic environmental fluctuations (Krishnan et al., 2023). Cultures followed a daily dilution regime with a prescribed volume of culture (60% unless otherwise noted) dilution occurring 1 h before sunset. This daily dilution allows cultures to reach a steady-state density at the same time each day. Samples were taken from the dilutions performed. Samples were collected for 7–9 consecutive days for each independent run.

### 2.4 Chlorophyll estimation by spectrophotometry, cell count and biomass quantification

Total Chl was estimated by spectrophotometric assays using a dichromatic calculation at wavelengths 652 nm and 665 nm as described (Porra, Thompson, and Kriedemann, 1989). Cell numbers were estimated using an Attune Nxt Flow Cytometer (Thermo Fischer Scientific, United States) equipped with a 20 mW 488 nm (blue) laser. Red fluorescence from Chl *a* was detected using a 695/40 nm emission filter. 100 μL of each sample was analyzed at a flow rate of 25 μL min^−1^. Voltages of the forward scatter, side scatter and red fluorescence detectors were set at 420, 300 and 440, respectively. Threshold was set on the forward scatter at 25,000 and Chl fluorescence was used to gate cells (values >10,000). Biomass quantification of ash-free dry weight (AFDW) was previously performed as previously described (Weissman et al., 2018) using glass fiber filters (TCLP 0.7, PALL, United States).

### 2.5 Pigments extraction and identification/quantification by HPLC

Pigments were extracted and quantified by HPLC as described previously (Cano et al., 2021) with some modifications. Briefly, *Picochlorum celeri* cells were rapidly deposited after sampling onto a GF/F™ filter (Whatman™ 25 mm 1822–021, Cytiva, United Kingdom), and immediately placed in a screw-cap tube and flushed with high-pressure N_2_ to remove air. The vial was capped tightly and stored at −80°C. Extraction was performed with 1 mL of 80% methanol/20% acetone using 0.1 mm glass beads and processing in a bead-beater in the dark. Samples were carefully kept away from direct light and maintained in the dark, on ice, during and after the extraction process to avoid pigment degradation. After centrifugation (18,000*g*/3 min/4°C), 500 µL of the supernatant was filtered (0.2 µm pore size 13 mm diameter Acrodisk^®^ Syringe Filters 4,423, Pall, United States) into a 2 mL HPLC glass vial. An additional 500 µL of the 80% methanol/20% acetone mix was pushed through the filter to desorb any remaining pigments from the filter surface which was further extracted by pushing through 30 mL of air to recover all remaining liquid. The headspace of the HPLC vial was flushed with N_2_ before closing. Sample vials were kept refrigerated in the dark at 4°C in the HPLC autosampler until analysis. Pigments were identified and quantified using a HPLC system (Surveyor SRVYR, Thermo Fischer Scientific, United States) equipped with a bonded silica C30 column (YMC Carotenoid 3 µm CT99S03-2546WT, YMC America, United States) maintained at 25°C, preceded by a guard column (YMC guard cartridge 3 µm CT99S03-0204GC, YMC America, United States) at room temperature. The following gradient was used to elute pigments: solvent A, methanol/methyl tert-butyl ether (MTBE)/water (81/15/4); solvent B, methanol/MTBE/water (58/38/4); 0%–100% B (0–50 min). Injection volumes were 25 µL.

### 2.6 Photosynthetic characterization

*In vivo* O_2_-photoproduction rates were determined using a custom-built Pt-Ag/AgCl polarographic electrode system (ALGi, United States) as described previously (Weissman et al., 2018; Cano et al., 2021). Briefly, 1.5 mL of sampled cells (at the daily harvest time happening 1 h before sunset) were diluted (0.6–0.7 μg mL^−1^) and purged with 99% He/1% CO_2_ followed by the addition of 6 μL 1 M potassium bicarbonate to avoid any CO_2_ limitation during the assay. 1 mL of the sample was then rapidly injected into the glass sample cell and sealed. Light was applied for 3 min after an initial 5 min dark period. To acquire photosynthesis-irradiance (PI) curves, light with increasing intensities (Luxeon III Star, Lumileds, United States) was applied for 3 min illumination increments, followed by 3 min darkness. Light intensities used were 25, 50, 75, 100, 125, 150, 250, 500, 1,000, 1,500, 2,000 µmoles PAR m^−2^ s^−1^. Net O_2_-production rates were measured by the slope of the linear fit of the inner 50% of the illumination periods. The temperature was maintained at 33°C throughout the experiment. Electrodes were calibrated before each measurement using atmospherically equilibrated growth medium and deoxygenated medium.

77K emission spectra were obtained from cultures sampled from the ALGiSIM at the daily dilution time. Samples were suspended at a concentration of 5 μg mL^−1^ of total Chl (Horiba, Japan), flash frozen in liquid N_2_, and dark-adapted for 5 min before collecting emission spectra (650 nm–800 nm)) after excitation at 440 nm. Spectra were normalized to the maximum absorption peak at 688 nm, corresponding to the PSII core [CP47/CP43 (Lamb, Røkke, and Hohmann-Marriott, 2018)]. Due to the presence of an interfering peak at 722 nm from the media, a blank spectrum with “media only” was also obtained and further subtracted from all spectra before normalization.

PAR-to-biomass conversion efficiency is defined as the energy stored in biomass divided by the total energy supplied in light. PAR insolation (mol photons m^−2^ d^−1^) was calculated by integrating the light curve (Cano et al., 2021) over the entire day. This was multiplied by 217 kJ mol^−1^ photons (PAR) to get the effective supplied energy. An average density of 23 kJ g^−1^ was used to calculate the energy stored as algal biomass each day.

Fv/Fm and functional cross-section of PSII values were obtained from fluorescence kinetics and the optical absorption cross section were determined using an integrating sphere as described previously (Weissman et al., 2018).

## 3 Results

### 3.1 Construction and pigment composition analysis of WT, CAO, LCYe and CAO/LCYe strains

**FIGURE 1:**
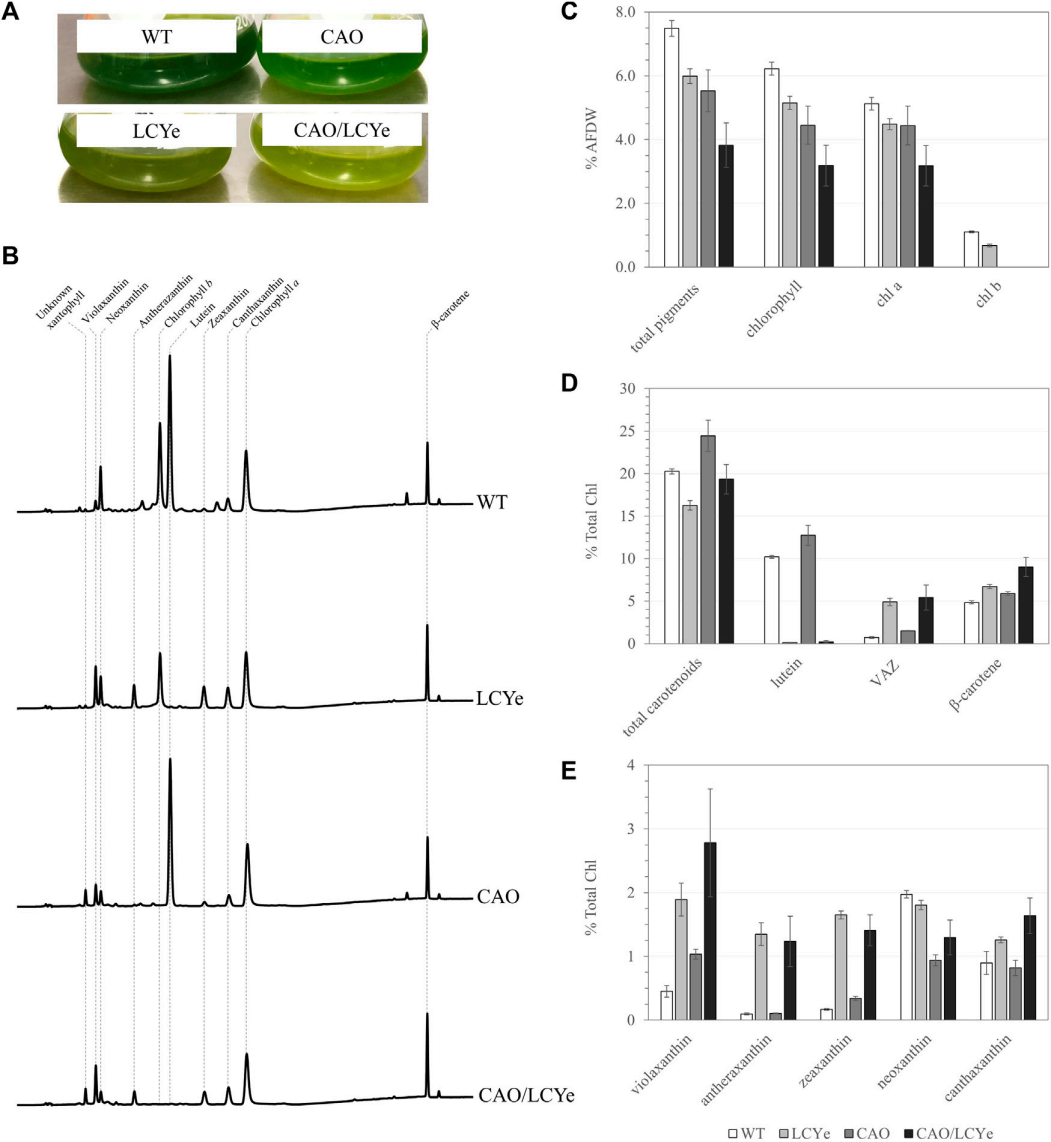
Pigment composition of the WT, LCYe, CAO and CAO/LCYe strains. **(A)** Visual depigmentation of the modified strains in liquid culture. **(B)** Chromatogram of HPLC pigment analysis **(C)** Pigment composition in %AFDW. **(D,E)** Detailed carotenoid composition. Each data point represents the average and standard deviation of 5 replicates.

Multiple knock-out mutants of the *CAO* gene were generated using RNP-mediated transformation of WT *Picochlorum celeri* and a linearized selection marker conferring resistance to CNAT (Supplementary Figure S1A). Complete knockout clones of the *CAO* gene were visually detected due to a depigmented phenotype on agar plates (Supplementary Figure S2) as well as in liquid culture (Figure 1A). All demonstrated a similar pigment composition (Figure 1B) lacking Chl *b*, confirming the link between the phenotype observed and the absence of *CAO* function. Details about screening can be found in Supplementary Figure S2. We could not amplify a WT sized amplicon of the *CAO* gene from the genome of clone M33 and could not detect Chl *b*, indicating a successful disruption of both copies of the native *cao* gene; M33 was chosen as the reference chlorophyll *b*-deficient strain (CAO). Similarly, we isolated multiple strains lacking lutein. Among the four clones tested on HPLC, three of them (M5, M6, M23) did not show any detectable levels of lutein and had a clear difference in pigmentation observed in liquid culture (Figure 1A) that was not easily detected on agar plates (Supplementary Figure S3). Indeed, pigmentation varies with growth conditions (growth phase, light intensity, media, nutrients, etc.), which are significantly different between liquid cultures and plates. All lutein-deficient strains tested using HPLC had a similar pigment composition to the reference lutein-deficient strain M5 (LCYe) used in the rest of the study (Figure 1B). Details about screening can be found in Supplementary Figure S3. We observed a high level of target-specific events for both *CAO* and *LCYe*, suggesting that those targets are amenable to disruption. CAO M33 was used as the background strain to generate the double deletion strain (CAO/LCYe) using the same sgRNA targeting *LCYe* and a linearized selection marker selecting for resistance to phleomycin (Supplementary Figure S1B). We obtained 3 clones with a non-functional *lcye* as suggested by the absence of lutein detected by HPLC and observed a similar pigment composition among them (data not shown). M2 was chosen as the reference *cao*/*lcye* strain. Details about screening can be found in Supplementary Figure S4.

In order to characterize the effect of the mutations on the pigment content and photosynthetic features under dense culture conditions, all four strains were grown in the ALGiSIM solar-simulating automated photobioreactors. All cultures were maintained axenic and algal strain purity was monitored throughout the campaign by their distinct whole cell UV-visible absorption spectrum (Supplementary Figure S5). We observed lower steady-state densities and productivities for the CAO and CAO/LCYe strains at a 60% daily dilution as compared to the WT (data not shown) indicating a deleterious effect from complete removal of Chl *b*. To assess their pigment composition, we instead performed single daily dilutions of 50% and 40% for CAO and CAO/LCYe, respectively, and 60% for WT and LCYe, in order to achieve similar light transmittance (measured with a light meter across the bottle) at the time of sampling (see Supplementary Figure S6; Supplementary Table S2) for fair comparison of the effect of diel irradiance. All three modified strains showed significant total pigment reduction compared to the WT: ∼20% for LCYe, ∼26% for CAO and up to ∼49% for CAO/LCYe (Figure 1C). In the complete absence of Chl *b*, the CAO strain showed ∼29% reduction in total Chl and ∼13% reduction in Chl *a*, as well as ∼14% reduction in carotenoids levels (Figure 1D). Interestingly, the LCYe strain not only had ∼34% reduction in total carotenoids but also ∼17% reduction in total chlorophyll compared to the WT strain, with ∼13% less Chl *a* and ∼39% less Chl *b*, leading to the higher Chl *a*/*b* ratio of 6.7 vs. 4.5 for the WT strain (Table 1) indicating a reduction in the peripheral antenna in this strain. While β-carotene (which has an essential photoprotective role in the reaction center) and canthaxanthin remain unchanged overall, we observed an increase in the total VAZ pool (violaxanthin, antherazanthin and zeaxanthin) in lutein-deficient strains (Figure 1E).

**TABLE 1:** Productivities of WT and LCYe strains in diel conditions. *n* = 24 sampling days corresponding to 3 biological replicates.

	WT	LCYe
Daily dilution (vol.)	60%	60%
AFDW (mg L^-1^)	1,050 ± 42	1,096 ± 42
OD_750_	4.5 ± 0.2	4.3 ± 0.1
Total Chl (µg mL^-1^)	46.8 ± 1.2	36.2 ± 1.0
Total Chl/AFDW (%)	4.5 ± 0.2	3.3 ± 0.2
Chl *a*/*b* (spectrophotometry)	4.3 ± 0.3	6.3 ± 0.3
Chl *a*/*b* (HPLC)	4.6 ± 0.3	6.7 ± 0.3
Specific growth rate (µ, day^-1^)	0.92 ± 0.04	0.92 ± 0.05
Biomass productivity (g m^-2^ day^-1^)	41.8 ± 2.5	43.9 ± 2.6
PAR-to-biomass conversion efficiency (%)	6.2 ± 0.4	6.6 ± 0.4

### 3.2 LCYe has a higher maximum quantum efficiency at lower light intensities

**FIGURE 2:**
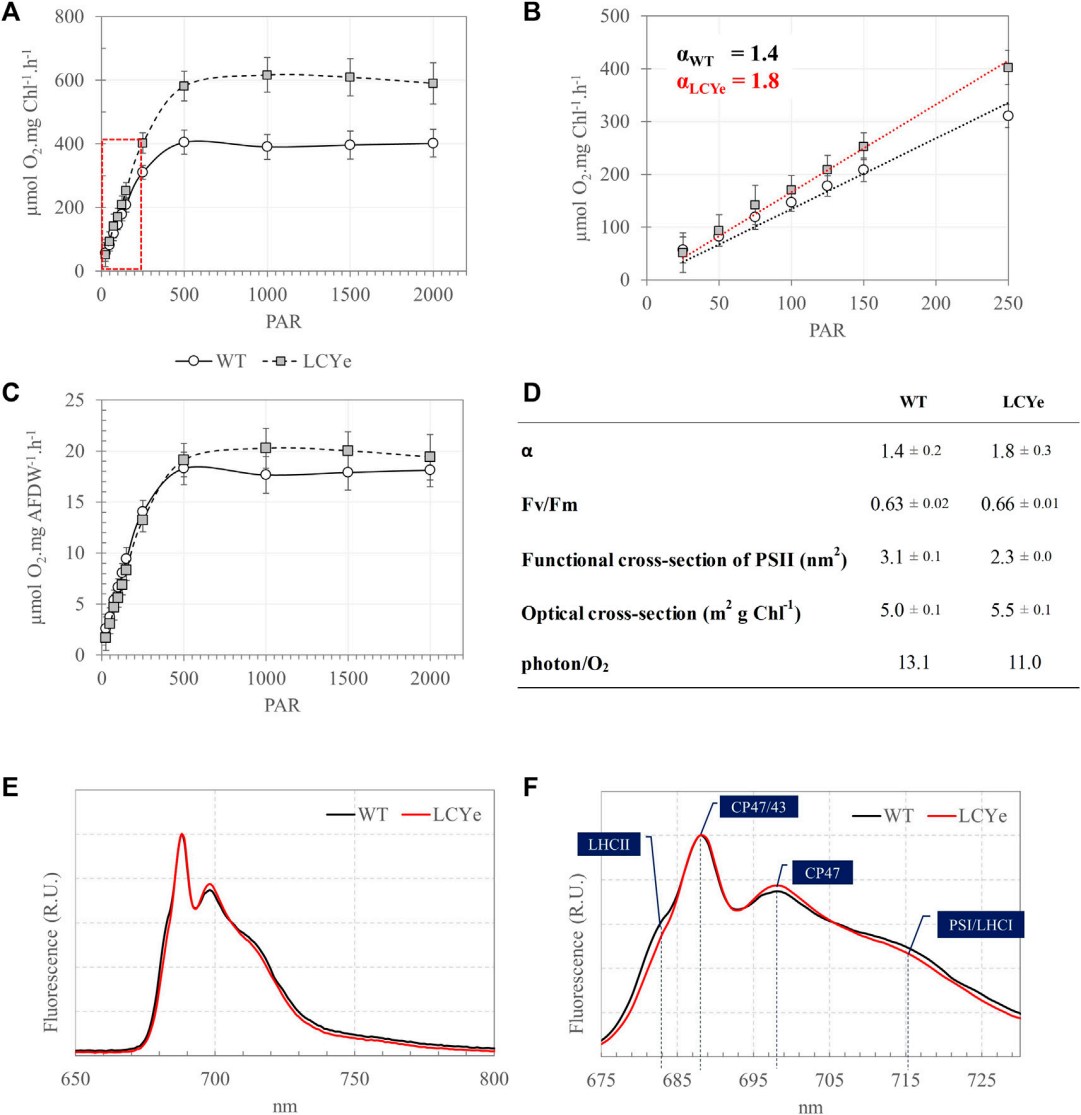
Photosynthetic features of the WT and LCYe strains. **(A)** Photosynthesis-Irradiance (PI) response curves of the WT and LCYe strains normalized per chlorophyll. **(B)** Close-up of the linear section of the PI curve delineated in red in A and determination of the slope α of the linear regression. **(C)** PI curves of the WT and LCYe strains normalized per AFDW. **(A–C)** *n* = 14 sampling days, over 3 biological replicates. **(D)** Photosynthetic parameters: α, Fv/Fm, functional and optical absorption cross-section, and quantum requirement (photons required per O_2_ evolved). **(E)** 77K emission spectra of the WT (black) and LCYe (red) strains. **(F)** Close-up of the spectrum shown in **(E)**. **(E,F)** *n* = 4 sampling days; 2 biological replicates.

Given an obvious impairment on growth of the CAO and CAO/LCYe strains relative to the WT, further analyses were carried out with the promising LCYe strain only. Samples were collected at sampling time to perform biomass and photosynthetic assays over a period of 7–9 days in each independent run. To determine the effect of depigmentation on O_2_ production, we obtained Photosynthesis-Irradiance (PI) response curves (Figure 2A). At lower light intensities (<150 µmole PAR m^2^ s^−1^), the photosynthetic rate increases linearly with increasing irradiance and the rate of electron transport from water to CO_2_ along the photosynthetic chain is limited by the rate of photon absorption. At higher irradiance (>150 µmole PAR m^2^ s^−1^), the photosynthetic rate increases non-linearly and reaches a maximum rate (P_max_) above which all excess energy is dissipated. The slope (α) of the linear regression of net O_2_ [production-consumption] vs. irradiance in the low light portion of the curve (25–250 µmole PAR m^2^ s^−1^) was 1.4 ± 0.2 and 1.8 ± 0.3 for WT and LCYe (two-tailed *t*-test, *p*-value = 0.0001), respectively, implying that the product of quantum yield of photosynthesis and the optical absorption cross-section of the strain was significantly higher in LCYe vs. WT (Figures 2B, D). Saturation occurred at irradiance higher than 500 µmole PAR m^2^ s^−1^ for both strains. As expected from the lower chlorophyll content per AFDW measured in LCYe (3.3% vs. 4.5% in the WT, Table 1), we obtained a higher *P*_max_ on a per chlorophyll basis in LCYe vs. WT (∼600 µmol O_2_ mg Chl^−1^ h^−1^ vs. ∼ 400 µmol O_2_ mg Chl^−1^ h^−1^ in the WT). Interestingly, *P*_max_ was also higher for LCYe when normalizing the O_2_ production per AFDW (Figure 2C): *P*_max_ was ∼20 µmol O_2_ mg AFDW^−1^ h^−1^ in LCYe and ∼18 µmol O_2_ mg AFDW^−1^ h^−1^ in the WT (two tailed *t*-test gave *p*-values of 0.004 and 0.022 for the time points 1500 and 2000 PAR, respectively). Maximum quantum yield of PSII (Fv/Fm) were similar (Figure 2D). We measured a decreased functional absorption cross-section of PSII suggesting a smaller PSII antenna size and a slightly higher optical absorption cross-section for LCYe compared to the WT, as expected from the pigment packing effect. Quantum requirement (photons needed per O_2_ generated, the inverse of quantum efficiency) calculated using α from Figure 2B and the measured optical absorption cross section suggested that quantum requirement is lower for LCYe, with ∼11.0 photons needed per O_2_ evolved, vs. ∼13.1 for the WT (Figure 2D). Taken together, these results suggest that LCYe has an increased photosynthetic efficiency compared to the WT in the conditions tested. Comparison of 77K emission spectra of WT and LCYe strains indicates a reduction in fluorescence yield associated with functional LHCII in the lutein-less mutant (F680 nm) (Figures 2E, F). Studies in *Arabidopsis thaliana* have shown that the *lut2.1* mutant lacking lutein compensate by binding violaxanthin in sites L1 and L2 of LHC proteins. This substitution reduces the antenna size and prevents the trimerization of LHCII complexes (Dall'Osto, Bressan, and Bassi, 2015). We hypothesize that a similar phenotype occurs in the LCYe strain of *Picochlorum celeri*.

### 3.3 LCYe can reach daily productivities higher than WT

**FIGURE 3:**
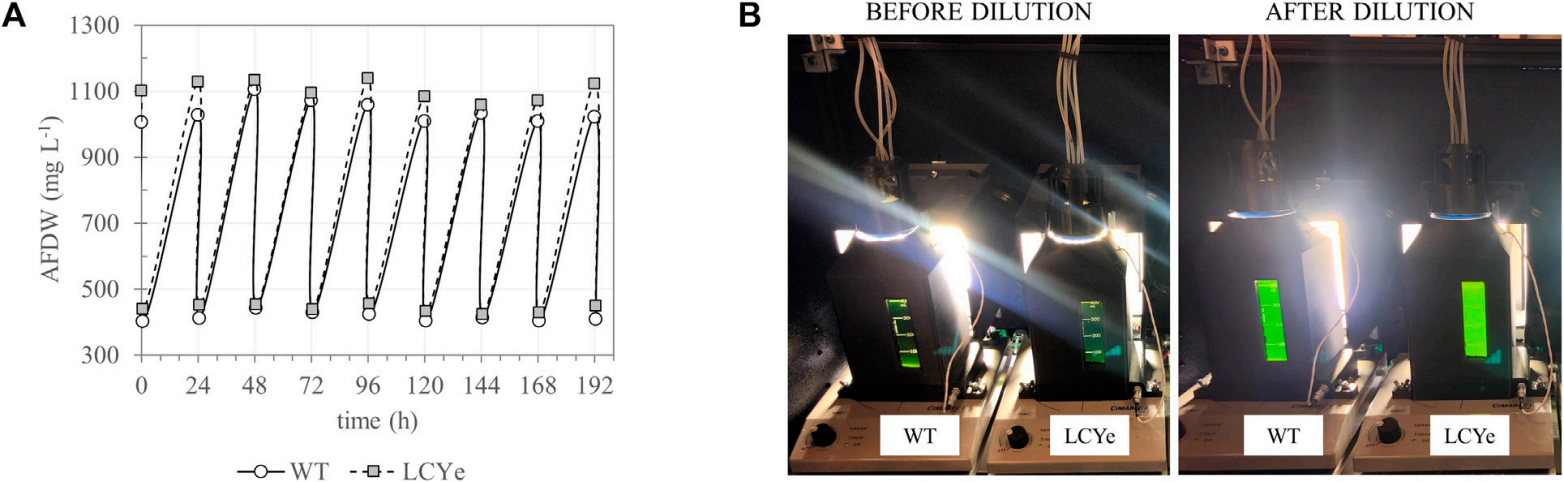
Daily harvest of WT and LCYe cultures in a custom-built solar-simulating automated photobioreactor under a diel regimen. **(A)** Culture density fluctuation over the course of 9 consecutive days (time 0 indicates the first harvest day after reaching steady-state). Cultures were harvested for AFDW estimation and other downstream assays 1 h before sunset. **(B)** Photobioreactor setup before (left) and after (right) the daily dilution occurring 1 h before sunset.

In addition to higher photosynthetic efficiency in subsaturating light, biomass measurements in diel conditions indicated higher biomass productivities for LCYe vs. WT. While both strains had similar specific growth rate (0.038 h^−1^ or 0.92 days^−1^) (Table 1), LCYe cultures reached a higher average steady-state density of 1,096 ± 42 mg L^−1^ (corresponding to a cell concentration of ∼ 2.2 × 10^5^ cell μL^−1^) vs. 1,050 ± 42 mg L^−1^ (corresponding to a cell concentration ∼ 1.9 × 10^5^ cell μL^−1^) in the WT (two tailed *t*-test gave *p*-value of 0.00033, Table 1; see also Figure 3 showing the steady state reached by both cultures before the daily harvest and post-60% dilution during 9 consecutive days). The WT average daily productivity was 41.8 ± 2.5 g m^−2^ day^−1^ while the LCYe was at 43.9 ± 2.6 g m^−2^ day^−1^ (two tailed *t*-test gave *p*-value of 0.018) over 3 independent runs (3 biological replicates) corresponding to 24 sampling days, representing a 5% increase in biomass productivity compared to the WT (Table 1). Correspondingly, the PAR-to-biomass conversion efficiency calculated was ∼6.6% for LCYe vs. ∼6.2% for the WT.

## 4 Discussion

Our preliminary results suggest that lutein is not necessary in controlled diel conditions to sustain high productivities in *Picochlorum celeri*. The lutein-deficient (LCYe) strain may even have a slight advantage over the WT strain in the conditions tested, with a moderate 5% increase in daily biomass productivity. This strategy could be applied to engineered strains with already reduced levels of chlorophyll to further decrease the absorption cross-section. While it appears that lutein could be removed without penalty in conditions simulating the light profile observed in an outdoor pond in *Picochlorum celeri*, the presence of chlorophyll *b* seems to be critical and complete removal should not be performed. These observations validate the potential of the pigment reduction strategy to improve biomass productivity in high-density cultures and suggest potential benefits to additional engineering layers targeting the lutein pigment specifically on existing strains that already show some improvement.

Non-photochemical quenching (NPQ) processes play a crucial role in protecting photosynthesis by quenching singlet-excited chlorophyll molecules, preventing photodamage by dissipating excess energy as heat (Müller et al., 2001). The xanthophyll cycle operated by the VAZ pool (violaxanthin, antherazanthin and zeaxanthin) is one of the major mechanisms involved in energy-dependent quenching (qE), the most common and well-understood type of NPQ. It plays a key role in protecting the photosynthetic apparatus, especially under varying light conditions, enhancing adaptation to fluctuating light, and improving stress tolerance. Lutein has also been implicated in NPQ, as suggested by several studies showing how its removal decreases qE (Niyogi, Bjorkman, and Grossman, 1997; Pogson et al., 1998). Double mutants of *Chlamydomonas* or *Arabidopsis* that lack lutein and zeaxanthin are totally devoid of any qE and are very sensitive to high light (Niyogi, Bjorkman, and Grossman, 1997; Niyogi et al., 2001), while an increase in lutein was linked to an increase in the rate of qE induction (Pogson and Rissler, 2000; Li et al., 2009). We observed an increase in the total xanthophyll pool (violaxanthin, antherazanthin and zeaxanthin) in lutein-deficient strains, which could help compensate for the lack of lutein to sustain photoprotective mechanisms during the peak of the solar day. In natural environments, photosynthetic organisms are exposed to ever changing conditions (varying intensities of sunlight, fluctuating incoming irradiance due to cloud coverage, damaging ultraviolet radiation, temperature variations, etc.) that were not explored in this study. Lutein, as a carotenoid pigment, absorbs excess light energy during photosynthesis, thereby preventing the formation of reactive oxygen species and minimizing photodamage to the photosynthetic apparatus. While our automated photobioreactor can mimick any solar day, the multiple factors that can affect outdoor cultivation were beyond the scope of the current project and are the focus of ongoing studies. It is likely that the repetition of the exact same light curve over our 7–9 days experimental runs helps create a relatively steady culture that is not challenged by simulated cloud cover or other stochastic outdoor parameters where lutein may be important for protection. The light curve represents a simulated day characteristic of an average summer day in Mesa, Arizona, excluding cloud cover, fog, rain, or other dynamic environmental fluctuations. We acknowledge that the aerial productivities reported in this study are confined to the specific conditions of this study and represent values extrapolated from volumetric productivities obtained from idealized days that are unlikely to extend to stochastic outdoor conditions. Future investigations will delve into the effects of lutein under fluctuating environmental parameters. *Picochlorum celeri* has been studied in outdoor pond cultivation campaigns and found to be suitable mainly for warm seasons (Huesemann et al., 2023a; Huesemann et al., 2023b; Gao et al., 2023; McGowen et al., 2023). While we believe the lutein-deficient (LCYe) strain generated in this study might be of value during the summer season and should be tested for outdoor cultivation, significant limitations might arise in fluctuating light and temperature conditions, and particularly in cold weather when NPQ processes might be hampered (Manjre et al., 2022), making this strain not suitable for winter cultivation in cold climates. Beyond the pigment reduction strategy explored in this work, additional improvements will be needed to increase growth rates and biomass yields to make algae a viable solution for biofuels and specialty products generation that will include genetic engineering of algal physiology and metabolic networks, strain selection as well as optimization of cultivation techniques (Melis, Hidalgo Martinez, and Betterle, 2023).

## Data Availability

The original contributions presented in the study are included in the article/Supplementary Material, further inquiries can be directed to the corresponding author.
