# Supplementary material for: Cas9 deletion of lutein biosynthesis in the marine alga Picochlorum celeri reduces photosynthetic pigments while sustaining high biomass productivity
Source: Front Bioeng Biotechnol. 2024 Jan 11;11:1332461. doi: 10.3389/fbioe.2023.1332461 (PMC10808502; doi:10.3389/fbioe.2023.1332461)
Supplement: Supplementary file 1 [file Presentation1.pdf]

Supplemental Figure S1

Selection markers used for genome engineering in this study. A) Left, pGAPDHNAT marker, carrying the CNAT (nourseothricin) resistance cassette (Krishnan et al. 2020). Right, pGADHNAT marker linearized at the *Sca*I site. B) Left, pGAPDHble marker, carrying the phleomycin resistance cassette. Right, pGADHble marker linearized at the *Sca*I site. pGAPDH, native *Picochlorum celeri* promoter of the Glyceraldehyde-3-Phosphate Dehydrogenase gene (*gapdh*); tRBCS2, native *Picochlorum celeri* terminator of the small RubisCO subunit 2 gene (*rbcs2*). Internal primers to the ampicillin cassette used for screening are indicated (Amp_dwn in blue and Amp_up in red).

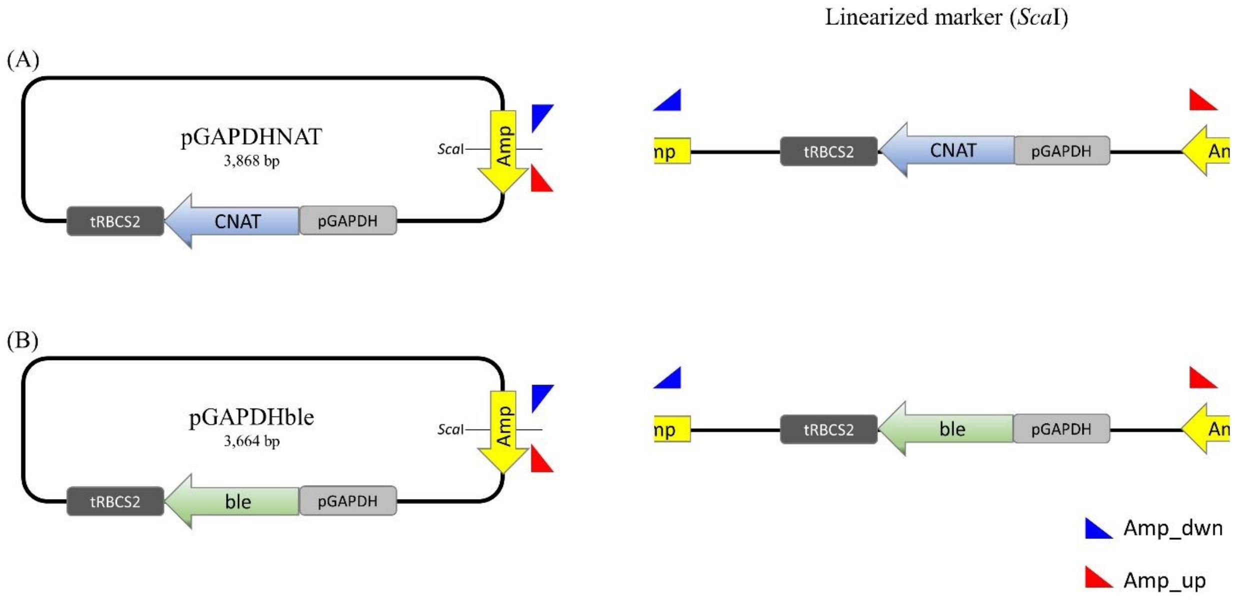

Supplemental Figure S2

Screening of RNP-*cao* WT *Picochlorum celeri.* A) Transformants restreaked on selection plate (f/4eU CNAT 75 µg mL^{-1}). B) Possible insertions of the linearized marker into the cut site of the *cao* gene following a RNP transformation. The WT-version of the *cao* gene is represented in the upper part of the figure, flanked by screening primers Cao_fwd/Cao_rev, resulting in a 2056 bp amplicon. The PAM site (pink triangle) and the targeted region of the sgRNA (black arrow) are indicated. The two possible insertion cases of the linearized marker are presented below, along with the position of all screening primers: insertion 1, resulting in a ~1000 bp amplicon with primers Amp_up/Cao_rev; insertion 2, resulting in a ~620 bp amplicion with primers Amp_dwn/Cao_rev. C) PCR screening of clones obtained from the RNP transformation targeting the *cao* gene in *Picochlorum celeri*. Panel I: amplification of the *cao* gene region (expected WT-size band of 2056 bp). Panel II, amplification overlapping the marker and the *cao* gene in case 1 shown in B. Panel III, amplification overlapping the marker and the *ca*o gene in case 2 shown in B. Clones that do not amplify the WT-size *cao* gene are highlighted in red. D) Sequencing of bands extracted from gel presented on Panel I in C. Mutants highlighted in red exhibit bases deletion near the PAM site.

We generated various strains lacking Chl *b* by targeting the *cao* gene using RNP-mediated transformation of the WT strain of *Picochlorum celeri* using a sgRNA targeting *cao* and a linearized selection marker selecting for resistance to CNAT (Supplemental Fig. S1A). We obtained ~100 clones out of which 48 were restreaked. 13/48 of the mutants (27 %) displayed a visually detectable depigmented phenotype on plate (Supplemental Fig. S2A) as well as in liquid culture (Figure 1A). 17 clones were screened by PCR and HPLC. Screening was performed considering the two possible insertions of the linearized marker (selecting for CNAT resistance) into the cut site of *cao* using primers flanking *cao* and primers overlapping the linearized selection marker and *cao* in both possible direction (Supplemental Fig. S2B). Interestingly, all clones tested amplified an amplicon indicating the insertion of the selection marker at the cut site in one or the other direction, suggesting a very high level of target-specific events (Supplemental Fig. S2C). 14/17 clones amplified an amplicon with a size corresponding to the WT version of the gene. Sequencing revealed that 9 out of those 14 clones had a conserved WT sequence, while 5/14 displayed deletion events (Supplemental Fig. S2D) and did not show the presence of Chl *b* in HPLC (Figure 1B). 3/17 clones did not amplify the WT-version of the *cao* gene (M1, M33, M38) and did not show any presence of Chl *b*, indicating a successful disruption of both copies of the native *cao* gene. A similar pigment composition (Figure 1B) was observed between all clones lacking Chl *b* (data not shown) confirming the link between the phenotype observed and the absence of *cao* function. Clones with a single KO allele that retained a WT-version of the gene did not show any pigment or growth phenotype (data not shown). We chose M33 as the CAO reference strain for further analysis.

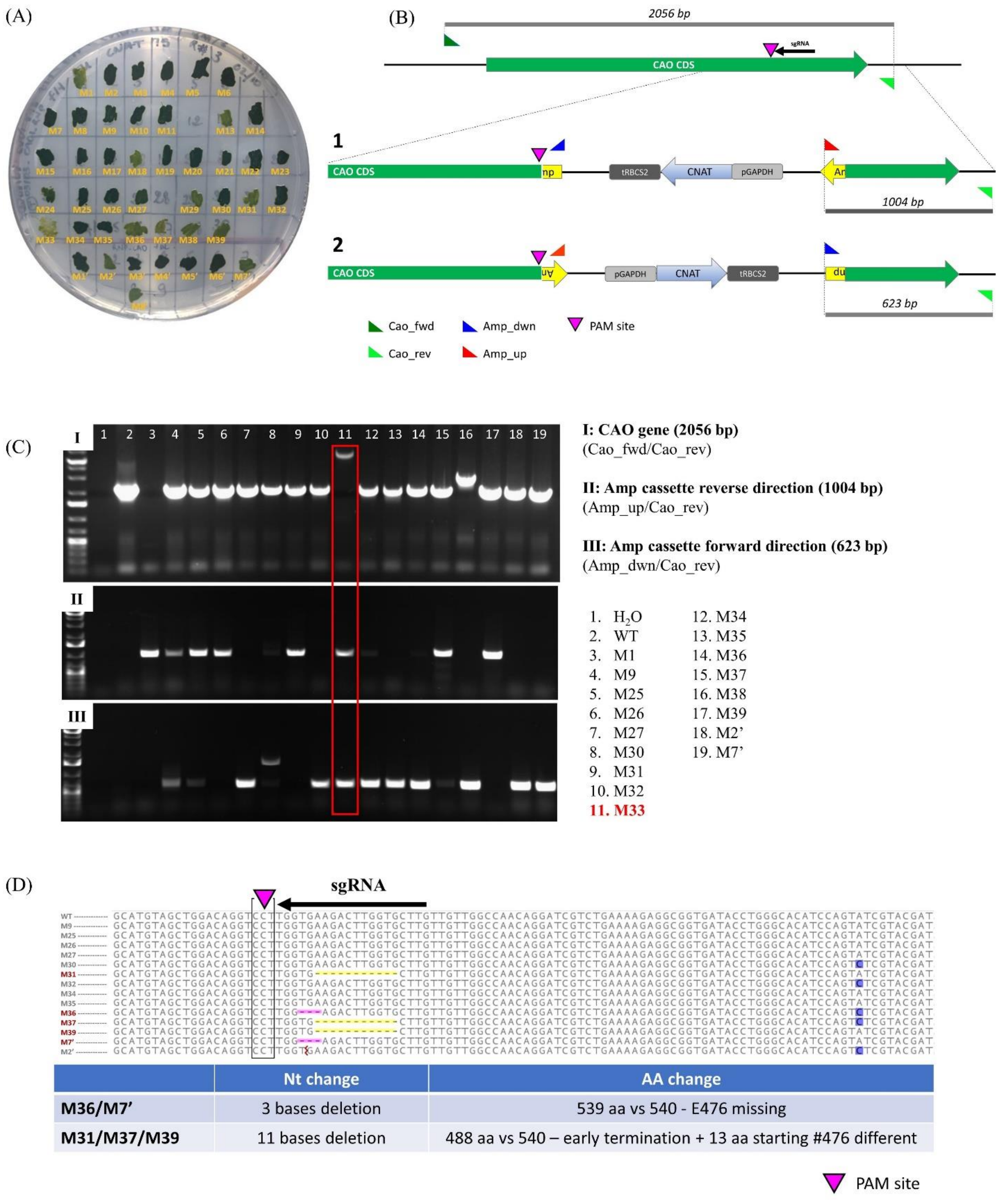

	Nt change	AA change
**M36/M7'**	3 bases deletion	539 aa vs 540 - E476 missing
**M31/M37/M39**	11 bases deletion	488 aa vs 540 – early termination + 13 aa starting #476 different

Supplemental Figure S3

Screening of RNP-*lcye* WT *Picochlorum celeri*. A) Transformants restreaked on selection plate (f/4eU CNAT 75 µg mL^{-1}). B) Possible insertions of the linearized marker into the cut site of the *lcye* gene following a RNP transformation. The WT-version of the *lcye* gene is represented in the upper part of the figure, flanked by screening primers Lcye_fwd/Lcye_rev, resulting in a 892 bp amplicon. The PAM site (pink triangle) and the targeted region of the sgRNA (black arrow) are indicated. The two possible insertion cases of the linearized marker are presented below, along with the position of all screening primers: insertion 1, resulting in a 773 bp amplicon with primers Amp_up/Lcye_rev; insertion 2, resulting in a 392 bp amplicion with primers Amp_dwn/Lcye_rev. C) PCR screening of clones obtained from the RNP transformation targeting the *lcye* gene in *Picochlorum celeri*. Panel I: amplification of the *lcye* gene region (expected WT-size band of 892 bp). Panel II, amplification overlapping the marker and the *lcye* gene, with a reverse insertion of the marker. Panel III, amplification overlapping the marker and the *lcye* gene, with a forward insertion of the marker. Sequencing of the bands extracted in panel I appear in the right column of the table. "X" indicates that there is no insertion of the selection marker detected. "✓ " indicates a sequencing identical to the native gene. D) Sequencing of PCR products bands extracted from the gel presented on Panel I in C. The nucleotide (Nt) sequence and resulting amino acid (AA) changes in the protein sequence are detailed in the table below.

We generated various strains lacking lutein by targeting the *lcye* gene using RNP-mediated transformation of the WT strain of *Picochlorum celeri* using a sgRNA targeting *lcye* and a linearized selection marker selecting for resistance to CNAT (Supplemental Fig. S1A). We obtained and restreaked 41 clones that did not show an apparent visual phenotype on plate (Supplemental Fig. S3A). 8 clones were screened by PCR (Supplemental Fig. S3B) and HPLC. PCR screening was performed using primers flanking *lcye* and primers overlapping the linearized marker (selecting for CNAT resistance) and *lcye* in both directions (Supplemental Fig. S3B). All 8 clones tested showed an amplicon with a size corresponding to the WT version of the gene (Supplemental Fig. S3C panel I). 7/8 clones exhibited an amplicon suggesting the insertion of the marker near the PAM site, 1 in one direction, 6 in the other (see Supplemental Fig. S3C panel II and III); unexpected sizes of the amplicon for some of them suggested a more complex insertion of fragment(s) of the linearized marker into the cut site. 4/8 clones presented modifications in the sequence of their WT-size band amplified resulting in various modifications, from potential amino acid substitutions to shorter products (Supplemental Fig. S3D). Among the four clones tested on HPLC, three of them (M5, M6, M23) did not show any presence of lutein and had a more obvious visual phenotype in liquid culture (Figure 1A). All lutein-deficient strains tested on HPLC had a similar pigment composition to the reference stain M5 used in the rest of the study (Figure 1B).

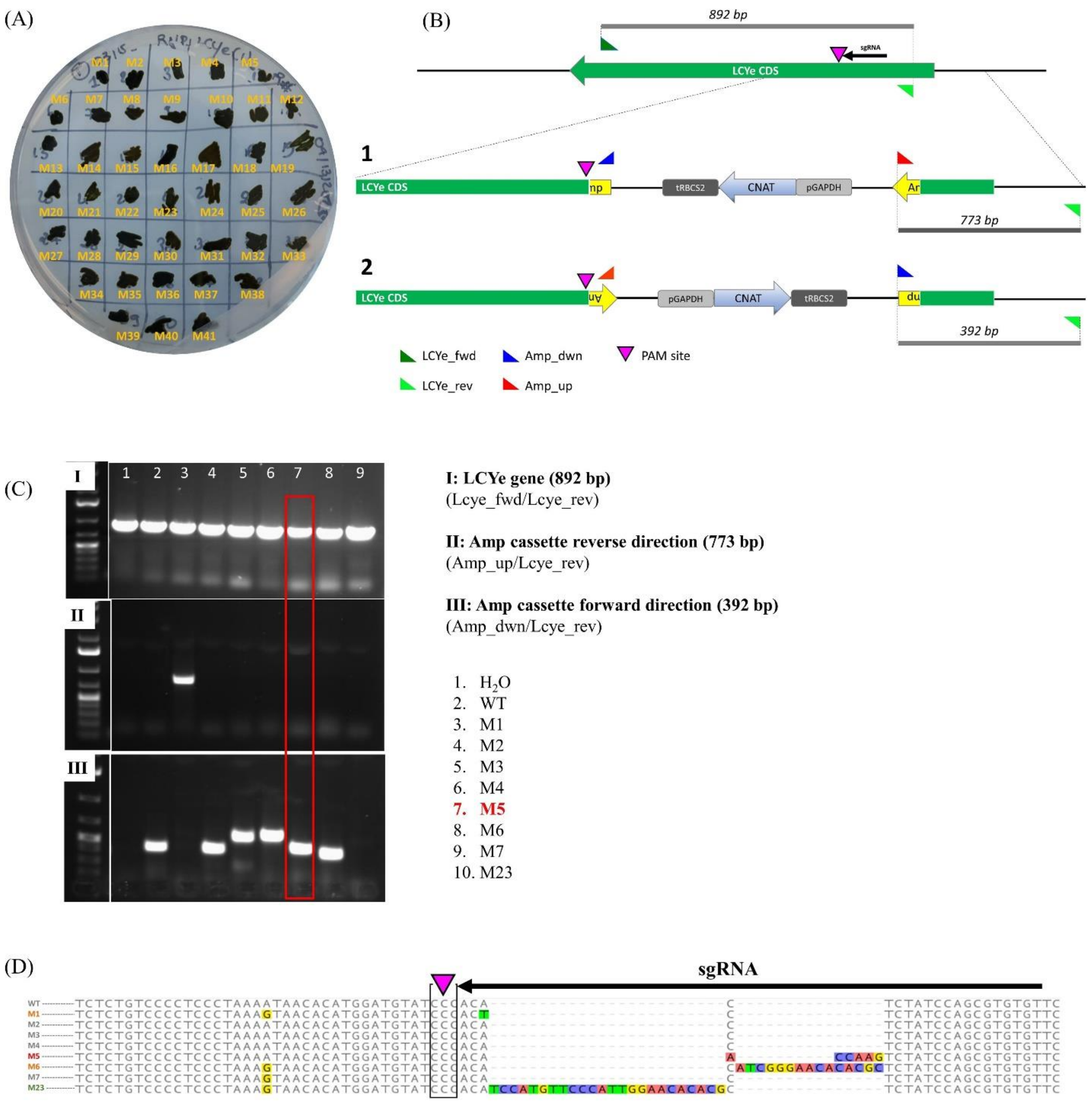

	Nt change	AA change
M1	Nt #383 A→T	Val128→Glu128
M5	1 nt change + 4 nts insert	152 aa vs 507
M6	15 nts insertion*	5 aa insert ACVPD (#128→#132)
M23	24 nts insertion*	8 aa insert ACVPMGTW +1 change (#128→#135, V136→M136)

PAM site

Supplemental Figure S4

Screening of RNP-*lcye* CAO (M33) strain. A) PCR screening of clones obtained from the RNP transformation targeting the *lcye* gene in the CAO (M33) strain. Panel I: amplification of the *lcye* gene region (expected WT-size band of 892 bp). Panel II, amplification overlapping the marker and the *lcye* gene, with a reverse insertion of the marker. Panel III, amplification overlapping the marker and the *lcye* gene, with a forward insertion of the marker. B) Sequencing of PCR products bands extracted from the gel presented on Panel I in A. The PAM site (pink triangle) and the targeted region of the sgRNA (black arrow) are indicated. The nucleotide (Nt) sequence and resulting amino acid (AA) changes in the protein sequence are detailed in the table below.

CAO (M33) was used as the background strain to generate the CAO/LCYe strain using the same sgRNA targeting *lcye* and a linearized selection marker selecting for resistance to phleomycin (Supplemental Fig. S1B). We obtained and restreaked 26 clones with a more accentuated depigmentation on plate and in liquid (Figure 1A). PCR screening of 8 of those clones revealed that 7/8 clones had at least one amplicon suggesting the insertion of the selection marker at the cut site (Supplemental Fig. S4A), confirming a high level of insertion in this specific cut site/locus. 2/7 clones that amplified a WT-size band had insertions of the linearized near the PAM site resulting in shorter products according to sequencing (Supplemental Fig. S4B) and that were non-functional, as suggested by the absence of lutein as detected by HPLC (data not shown). One clone (M2) amplified the selection marker in both directions with no detection of a WT-size band indicating a complete KO of the gene in both alleles and confirmed by the complete absence of lutein (Figure 1A & B). M2 was chosen as the reference CAO/LCYe strain.

(A)

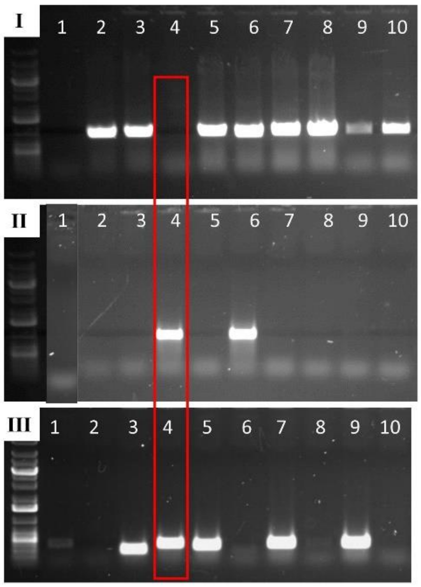

I: LCYe gene (892 bp)
(Lcye_fwd/Lcye_rev)

II: Amp cassette reverse direction (773 bp)
(Amp_up/Lcye_rev)

III: Amp cassette forward direction (392 bp)
(Amp_dwn/Lcye_rev)

1. H_2O
2. WT
3. M1
4. **M2**
5. M3
6. M4
7. M8
8. M10
9. M11
10. M12

(B)

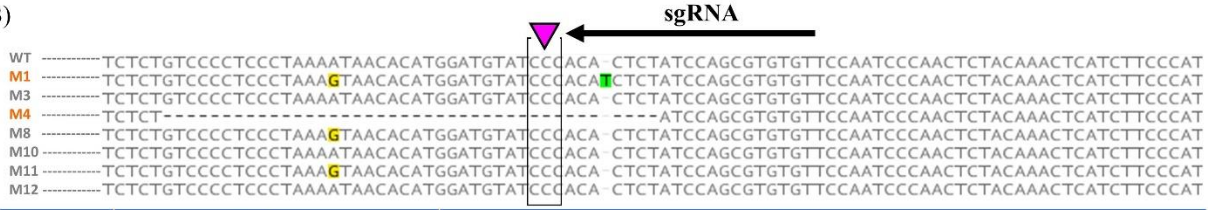

	Nt change	AA change
M1	1 nt insertion	138 aa vs 507
M4	41 nts deletion	136 aa vs 507

▼ PAM site

Supplemental Figure S5

UV-visible absorption spectra of WT, LCYe, CAO and CAO/LCYe cultures grown in photobioreactors.

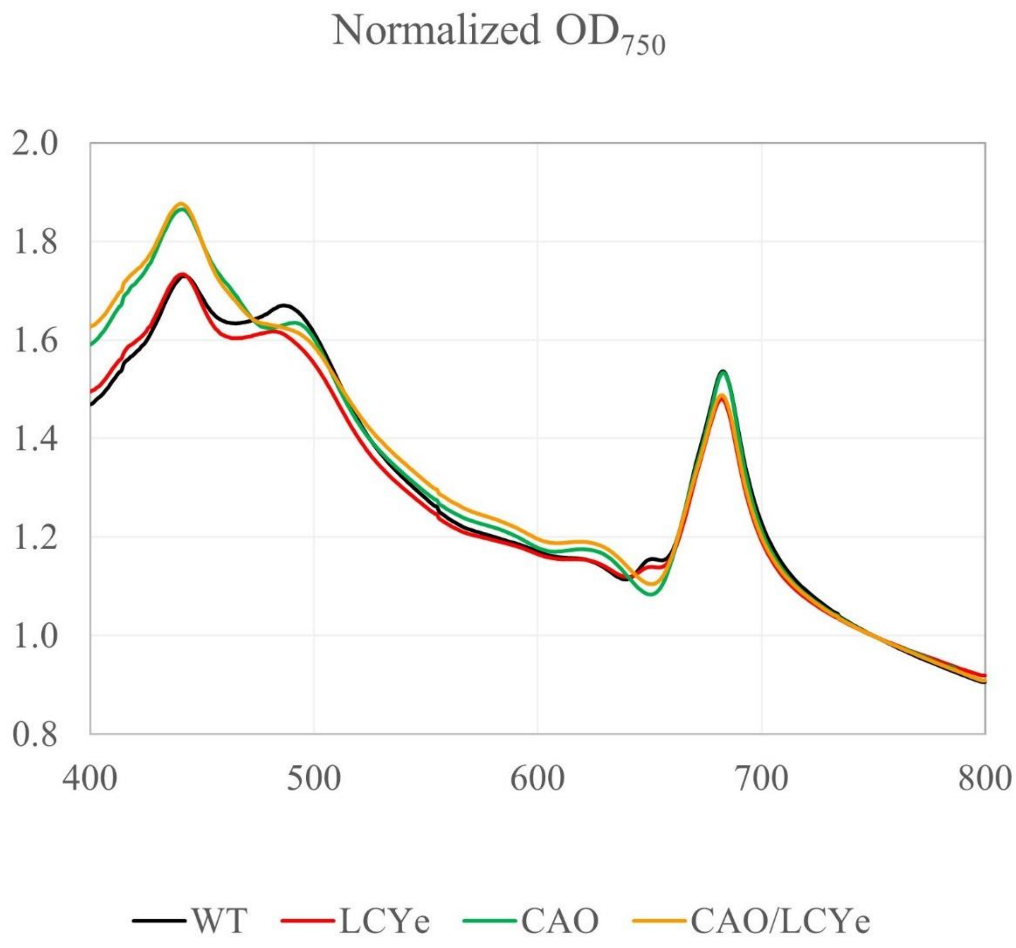

Supplemental Figure S6

Four stations custom-built solar-simulating and automated photobioreactor used in this study. Up: photobioreactor system before daily dilution, 1h before sunset. Down: system after daily dilution.

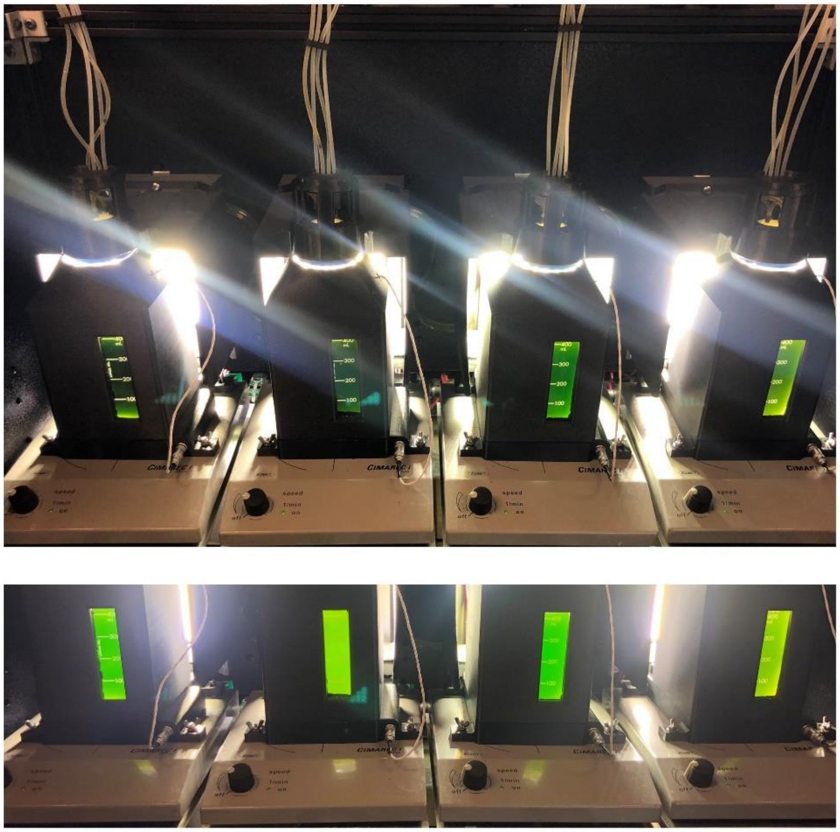

Supplemental Table S1

Primers used in this study.

Primer	Sequence
**sgRNA-*lcye***	TTCTAATACGACTCACTATAGacacacgctggatagagtgtGTTTTAGAGCTAGA
**sgRNA-*cao***	TTCTAATACGACTCACTATAGcaagcaccaagtcttcaccaGTTTTAGAGCTAGA
**Lcye_fwd**	CCTGACAATAGAATACCCCGTCGCTGG
**Lcye_rev**	GGCTGGTCTGTCACTGGCATCTGAATT
**Cao_fwd**	TGGACACGTGACACTCATCGAATCAGG
**Cao_rev**	ACCGCGAATTGACTGATTGCCAAGACT
**Amp_up**	CGTAACTCACGTTAAGGGATT
**Amp_dwn**	TTGCTCACCCAGAAACGCT

Supplemental Table S2

Productivities of CAO and CAO/LCYe strains in diel conditions. n=7 sampling days.

	CAO			CAO/LCYe		
**Daily dilution (vol.)**	50%			40%		
**AFDW (mg L^{-1})**	1205	±	73.6	1158	±	88
**OD_{750}**	4.7	±	0.1	4.1	±	0.3
**Total Chl (µg mL^{-1})**	35.7	±	2.4	25.5	±	2.1
**Total Chl/AFDW (%)**	3.0	±	0.1	2.2	±	0.1
**Chl *a*/*b* (spectrophotometry)**		-			-	
**Chl *a*/*b* (HPLC)**		-			-	
**Specific growth rate (µ, day^{-1})**	0.70	±	0.07	0.57	±	0.09
**Biomass productivity (g m^{-2} day^{-1})**	40.1	±	2.7	33.4	±	2.3
**PAR-to-biomass conversion efficiency (%)**	6.0			5.0		

Krishnan, Anagha, Melissa Cano, Tyson A. Burch, Joseph C. Weissman, and Matthew C. Posewitz. 2020. 'Genome editing using Cas9-RNA ribonucleoprotein complexes in the high-productivity marine alga Picochlorum celeri', *Algal Research*, 49: 101944.
